# Supplementary material for: Assessment of the Influence of 5-Fluorouracil on SMAD4 and TGFB1 Gene Expression, Apoptosis Induction and DNA Damage in Human Cell Lines
Source: Bioengineering (Basel). 2023 May 9;10(5):570. doi: 10.3390/bioengineering10050570 (PMC10215742; doi:10.3390/bioengineering10050570)
Supplement: Supplementary file 1 [file bioengineering-10-00570-s001.zip › bioengineering-2326845-supplementary.pdf]

bioengineering

MDPI

Supplementary Materials

Assessment of the Influence of 5-Fluorouracil on SMAD4 and TGFB1 Gene Expression, Apoptosis Induction and DNA Damage in Human Cell Lines

Agnieszka Wosiak [1,*], Dagmara Szmajda-Krygier [1], Jacek Pietrzak [1], Joanna Boncela [2] and Ewa Balcerczak [1]

1 Laboratory of Molecular Diagnostics and Pharmacogenomics, Department of Pharmaceutical Biochemistry and Molecular Diagnostics, Medical University of Lodz, 1 Muszynskiego, 90-151 Lodz, Poland
2 Institute of Medical Biology, Polish Academy of Science, 106 Lodowa, 93-232 Lodz, Poland
* Correspondence: agnieszka.wosiak@umed.lodz.pl; Tel.: +48-42-677-91-12

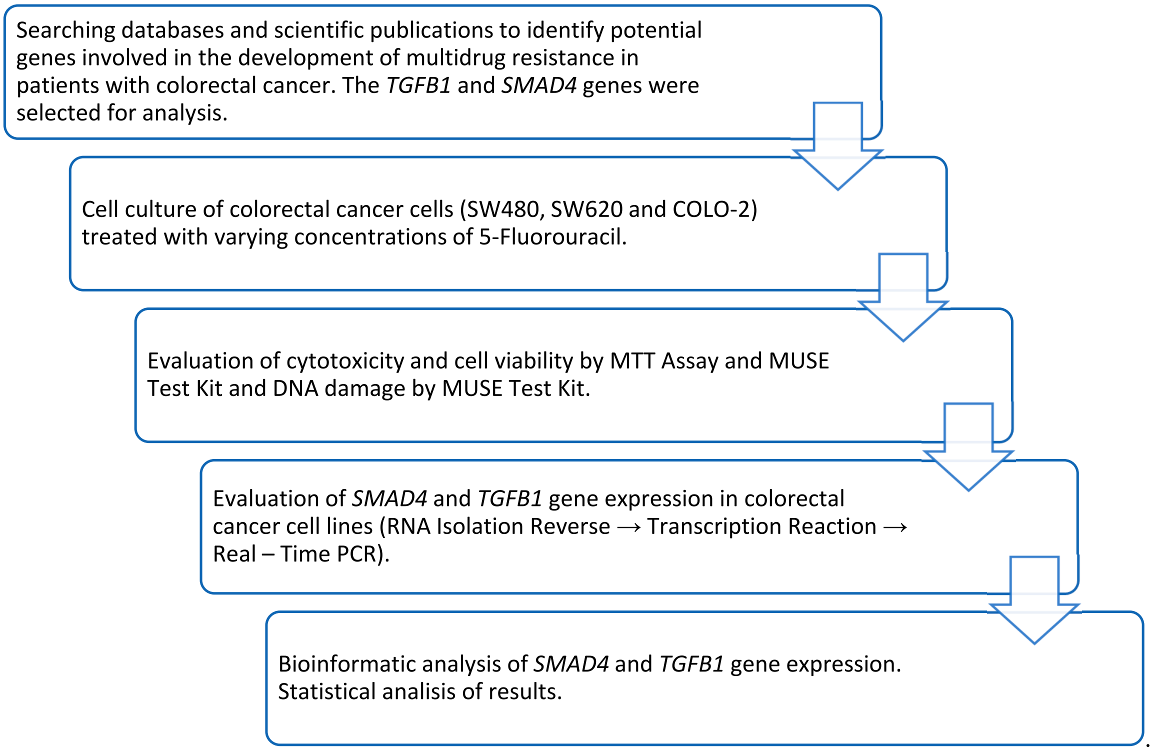

Figure S1. Research scheme.
